# Comment on "Factors influencing neonatal outcomes in twin pregnancies undergoing cesarean section: a cross-sectional study"

**DOI:** 10.1590/1806-9282.20231309

**Published:** 2024-04-22

**Authors:** Hua Tian, Jinjin Liu

**Affiliations:** 1 Shiyan Maternal and Child Health Hospital, Department of Maternity – Shiyan, China.

Dear Editor,

We read with great interest the recent study^1^ on neonatal outcomes in twin pregnancies undergoing cesarean section. The study's conclusion regarding the factors strongly associated with poor neonatal outcomes in twins delivered by cesarean section is particularly noteworthy. The findings highlight the significant impact of general anesthesia, emergency surgery, early gestational weeks, and birth weight below the 3rd weight percentile on neonatal well-being. Understanding these influential factors in twin pregnancies undergoing cesarean section is crucial for clinicians and healthcare providers. It not only aids in risk assessment but also allows for better-informed decision-making when planning and performing cesarean sections for twin pregnancies. The study's^1^ emphasis on these specific factors provides valuable insights that can contribute to improving the overall care and outcomes for both mothers and their neonates in twin pregnancies. However, we believe that the following aspects require further clarification.

First, while the study's^1^ conclusion indicates a strong association between poor neonatal outcomes and factors such as general anesthesia, emergency surgery, early gestational weeks, and birth weight below the 3rd weight percentile, it appears that the description provided in Table 3 of this study^1^ somehow contradicts these findings. Specifically, Table 3 suggests a correlation between birth weight below the 3rd weight percentile and an increased risk of mortality (OR=5.263, 95%CI [1.934, 14.321], p=0.001), indicating an adverse association with neonatal outcomes. However, in the case of early gestational weeks, the reported relationship with mortality is presented as OR=0.591, 95%CI [0.510, 0.684], p=0.000, which could be easily misinterpreted as a decrease in mortality associated with early gestational weeks. It is plausible that an inadvertent choice of reference group may have led to this discrepancy in the data presentation. It appears that early gestational weeks may have been mistakenly set as the reference group, with non-early gestational weeks as the observation group, resulting in OR=0.591, p=0.000. Similar issues seem to exist in the context of neonatal intensive care unit (NICU) and mechanical ventilation (MV) outcomes. Thus, to avoid any misinterpretation of the study's conclusions, it is imperative to clarify the data description further and ensure that the reported findings align with the study's intended analysis.

Second, we would like to emphasize another critical factor that plays a significant role in neonatal outcomes in twin pregnancies undergoing cesarean section, namely, anemia. Anemia is a multifaceted issue deserving careful consideration in the context of this study. A retrospective study^2^ involving 427 participants found that the anemic group had higher rates of low 1-minute Apgar scores (4.4% vs. 1.8%, p=0.028), perinatal death (1.9% vs. 0.2%, p=0.012), and NICU admissions (27.2% vs. 20.2%, p=0.017). It also discovered that after adopting oral iron therapy, the recovered group showed a lower NICU admission rate (13.5% vs. 30.3%, p=0.006), along with higher gestational week and birth weight (β, 0.954 week and β, 171.01 g; respectively). This evidence strongly suggests a correlation between anemia and neonatal outcomes in twin pregnancies and the presence of anemia is evidently associated with adverse neonatal outcomes. Although the current study^1^ provides some information about anemia, it does not explore the relationship between anemia and neonatal outcomes in twin pregnancies comprehensively. Given that anemia is a reversible pathological condition, further investigation into its connection with neonatal outcomes in twin pregnancies, along with the implementation of appropriate strategies to rectify anemia, has the potential to significantly improve neonatal outcomes in this context.
